# Social exclusion, corruption, recall of authorities, inequality and fiscal centralization: inducers of social conflict in Peru (2016–2023)

**DOI:** 10.3389/fsoc.2024.1419737

**Published:** 2024-06-07

**Authors:** Teófilo Lauracio Ticona, Mario Aurelio Coyla Zela, Jarol Teófilo Ramos Rojas, José Luis Morales Rocha, Genciana Serruto Medina, Nakaday Irazema Vargas Torres

**Affiliations:** ^1^Professional School of Accounting, Jose Carlos Mariátegui University, Moquegua, Peru; ^2^Professional School of Public Management and Social Development, National University of Moquegua, Moquegua, Peru

**Keywords:** subnational governments, social conflicts, inequality, social exclusion, fiscal centralism, corruption, recall

## Abstract

The objective of the article was to investigate the possible inducing factors that contributed to determine the frequency of social conflicts at the subnational level in Peru between 2016 and 2021, including income inequality, social exclusion, fiscal centralism, corruption and revocation of authorities, for which four regression models were built. Disaggregated official data from the 24 departments and the provinces of Lima and Callao were analyzed. Economic inequality was associated with the Gini coefficient. To establish the association between social conflict and the inducers, it was estimated using Spearman’s Rho correlation coefficient. Statistical calculation was also employed to appreciate the collinearity between the inducers. The results showed that the revocation of subnational authorities determines 42.5% of social conflict. On the other hand, corruption and fiscal centralism determine 28.5% of the perception of suffering social exclusion. Inequality and social conflict determined 21.8% of the relevance of the execution and quality of public spending by the national government in the regions. Sixty percent of social conflicts in Peru are of an environmental nature. The population that has declared the greatest discrimination corresponds to Puno (28%). 55.6% of those surveyed consider corruption to be one of the country’s main problems. Corruption and social exclusion have a negative impact on the effectiveness of economic results and promote social conflicts. Inefficient use of fiscal resources translates into low quality of services and diminished credibility of the national and subnational governments. This situation highlights the need to design public policies that reduce conflicts and promote adequate governance.

## 1 Introduction

Political and institutional instability has worsened since 2016 (Malamud and Núñez, 2020), Peru has had seven presidents, four of them vacated before the end of their term and has experienced two irregular dissolutions of Congress. This unusual phenomenon in democracies, however incipient they may be, is usually attributed to the absence of social bases and democratic practices in political parties, whose leaderships seek to retain power (Ponce, 2023). Its consequences are dire for the country’s governability, human rights violations are systematic, it transitions from a flawed democracy to a hybrid regime; after a slight recovery, GDP growth fell at the end of 2023 to −0.5% in 2023 (Velarde, 2023); Moody’s downgraded investment confidence in Peru from stable to negative; inflation increased; nearly 700,000 Peruvians moved from middle class to vulnerable, equal population from vulnerable to poor (Carrasco, 2023; Francke, 2023; Ramos, 2023).

National journalism and academia investigate and report very little on the impact of national political instability and the consequent conflicts in the regions and localities of the “interior of the country,” even about the massive “provincial” demonstrations in the capital of the Republic; rather, they reinforced the actors as victimizers or victims in the imaginary; they made visible the economic losses, but obviated or disguised the information on the causes, dynamics or the possibility of transforming it from crisis to convergence and sustainable development (Cornejo, 2023). It seems that the role of journalism and academia in understanding the social conflict that occurs mainly in the central and southern departments of the country has been postponed. Crises are also opportunities to change strategies that have fallen apart (Quispe, 2021). The empathy of the central government and its officials to the aspirations of these populations is necessary to make effective the process of regionalization and political, fiscal and administrative decentralization, initiated a few decades ago (Casaretto et al., 2015).

The undeniable peak of escalation of social conflict at the end of 2022, constitutes a clear questioning of the elites that govern the country through authorities and officials inclined to impose policies focused on the care of the private interests of the elite, to the detriment of the resolution of major national problems, respect for human rights and the rule of law, sustainable use of the environment, regional and local institutions, etc. (De Echave, 2023). However, this questioning is not a conjunctural issue, it is an endemic manifestation whose origins possibly predate the Republic, given the complex territoriality with which the country was built (Seminario and Palomino, 2020; Castillo et al., 2023).

Between 2020 and 2023, it was up to the National Government to resolve 63.7% of social conflicts, regional governments 9.8%, local governments 7% and others: 6.3% (Ombudsman’s Office, 2024); however, although the central government has institutions that are responsible for resolving them, these are not adequately or timely articulated with civil society, less sub-national, nor do they have a transformative approach or the vision of sustainability of the extractive industry, the most important source of conflict (Cordova and Garcia, 2022; Gonzales et al., 2023).

Reflecting on the peremptory exit from the political crisis of 2019, Paredes and Encinas (2020) said “it is important to prevent false optimism about the strength of democracy in Peru, …, it is unfeasible to know if it will be able to come out of a similar crisis at another time”; as indeed happened soon, at the end of 2022. It seems that the endemic Peruvian social conflict is a response, particularly from the sub-national population, which transcends economic models, political stores, public policies, etc.; therefore, it is opportune and pertinent to contribute to reflect on the factors that keep it in suspense. In our opinion, it is the actors themselves who are called upon to resolve them, but in an informed manner. In the current Peruvian social and political crisis, from a socio-historical perspective, there are structural elements such as the process of state centralization, the subordination of the ruling classes in Lima to the highland and jungle communities, neo-liberalization through authoritarian means, the exclusion through criminalization of indigenous communities, peasants and marginal neighborhoods from their participation in the country’s political life, among others (Mendoza, 2023).

In this article we intend to explore some inducers that possibly feed social conflicts at the sub-national level, such as inequality of resources and endogenous opportunities for human, economic, technological, institutional, etc. development (Varona and Gonzales, 2021); social exclusion, referring to practices “that prevent certain social groups from fully participating in the economic, social, political and cultural spheres” of the country (Pozo and Arbieto, 2021); fiscal centralism: a mechanism of hierarchization of territories and sub-national authorities, and the digitization of the Research Topic and execution of public spending by the central government (Escobar et al., 2021; Baylon and Quispe, 2022; Gonzáles, 2022); corruption: abuse of authority and public function to obtain private benefits, biasing public or administrative policies to divert public resources (Chaparro et al., 2021); and, the recall of authorities: “a fundamental right and at the same time a mechanism for citizen participation” (Salinas, 2022), but, frequently, a source of instability and social conflicts.

Conflicts within countries negatively affect economic and social outcomes in both the short and long term, which requires that public policies be designed to counteract the negative effects of conflict and mitigate the effects of the environment. A higher level of a country’s income also has less adverse effects on generating conflict, which requires better management of fiscal resources (Le et al., 2022).

Montes and Paschoal (2016) indicate that less corrupt countries provide a better quality of services and adopt better public policies in favor of citizens’ demands and have greater credibility. It also concludes that the most indebted governments are less efficient governments and countries with more democratic regimes have greater governmental effectiveness.

Therefore, the following questions arise: What are the most important drivers of social conflicts in the national and subnational governments? Is social exclusion, centralization of public finances and corruption in the exercise of public functions the most important factors that determine social conflicts? Is inequality in the allocation of resources to subnational governments, the low percentage of public budget execution, the revocation of authorities, and the credibility of politicians, factors that determine government instability in recent years?

## 2 Method

The research coverage corresponds to the 24 departments, plus the province of Lima (or Metropolitan Lima) and the Constitutional Province of Callao. Data disaggregated by department and provinces was obtained from the websites of the Ministry of Economy and Finance (MEF), the National Institute of Statistics and Informatics (INEI), the Ombudsman’s Office and others.

**Figure 1:**
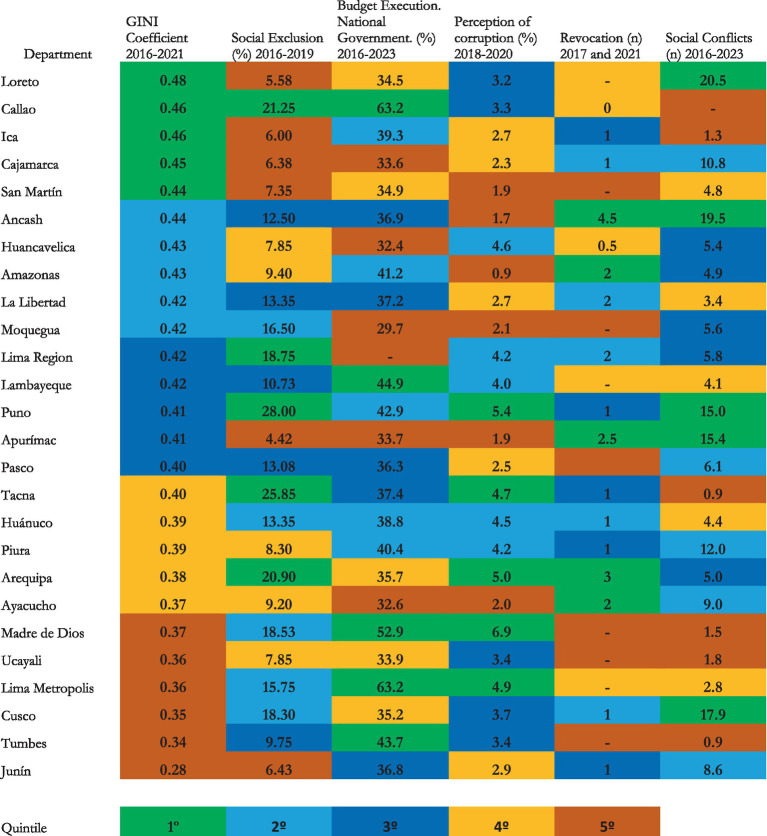
Peru: average of selected inducers of social conflict. Own elaboration with data from National Jury of Elections (2021), INEI (2023), Ministry of Economy and Finance (2024), Ombudsman’s Office (2024), and World Bank (2024).

Most economic inequality was associated with the Gini coefficient (World Bank, 2024); social exclusion with the percentage of the population aged 18 and over who reported having felt discriminated against (National Institute of Statistics and Informatics, 2023); financial centralism with the execution of the expenditure budget by levels of government, in the accrual phase (Ministry of Economy and Finance, 2024); corruption with the percentage of the population that reported having been demanded some additional payment for public services (INEI, 2023); data on the revocation of sub-national authorities obtained from the (National Jury of Elections, 2021), corresponding to the electoral processes of 2017 and 2021; and, social conflict was associated with the frequency of social conflicts by department (Ombudsman’s Office, 2024). The data has been organized by quintiles (see Figure 1).

After the Kolmogorov–Smirnov normality test, Spearman’s Rho correlation coefficient was estimated to establish the association between social conflict and the inducers that were considered to explain it and between them. The calculation of the statistic was also used to appreciate the co-linearity between the inducers. The correlation coefficients calculated were at most moderate, if not low and irrelevant. Nevertheless, two linear regression models were constructed to explain the relationship of conflict with the inducers that showed some level of correlation, a third model to explain social exclusion and a fourth one referring to fiscal centralism.

## 3 Result and discussion

The occurrence of the inducers chosen to explain the social conflicts was of gradual intensity and frequency in the Peruvian departments, in addition to the two provinces: Metropolitan Lima and Callao, which are generally equated to those for administrative and analysis purposes. This gradualness is presented from highest to lowest in Table 1, by quintiles.

**Table 1:** Spearman’s Rho correlation coefficients of subnational social conflict factors Peru, 2016–2023.

	Social conflict	GINI Coefficient	Social exclusion	Public finance centralism	Corruption	Revocation of authorities
Social conflict	1	0.296^**^	−0.131	−0.299^**^	−0.183	0.492^**^
GINI coefficient	0.296^**^	1	−0.127	−0.205^*^	−0.185	−0.05
Social exclusion	−0.131	−0.127	1	0.329^**^	0.492^**^	−0.14
Public finance centralism	−0.299^**^	−0.205^*^	0.329^**^	1	0.212	−0.345
Corruption	−0.183	−0.185	0.492^**^	0.212	1	
Revocation of authorities	0.492^**^	−0.05	−0.14	−0.345		1

Own elaboration with data from National Jury of Elections (2021), INEI (2023), Ministry of Economy and Finance (2024), Ombudsman’s Office (2024)), and World Bank (2024). *Correlation is significant at the 0.05 level (2-tailed). **Correlation is significant at the 0.01 level (2-tailed).

### 3.1 Subnational social conflict in Peru

Social conflict is inherent to human coexistence in society. Thinkers such as Marx, Dahrendorf, Durkheim, Weber, Parsons, Merton, etc., have tried to explain it through the theories of conflict and integration. Social conflict is a form of socialization, a product of interdependence; it occurs if two or more individual or group subjects, formal or informal, perceive that their objectives, interests, values, opinions, relationships, data, rights or obligations, etc., are incompatible; or that the facts or conditions of the physical or formal environment generate such incompatibility (Illera, 2022).

Social conflicts in sub-national spaces are mainly caused by the perception and/or verification of incompatible facts regarding the use of natural resources in a territorial area by the State, the business sector and civil society. Sixty percent of social conflicts in Peru are environmental in nature (Ombudsman’s Office, 2024) and are structural in nature, with roots in the vice regal era (Seminario and Palomino, 2020). Frequently, the extraction, processing and commercialization of natural resources, as of the other activities of production of goods and provision of services, are often accompanied by environmental impacts on sustainable development; among others, it generates inequalities in the family economy, in the access and enjoyment of environmental resources, breaks the structures of power and responsibilities, processes and culture of economic, social activities, etc., and care for the environment0020 (Fernández, 2020).

A second source of these conflicts is related to the centralist, authoritarian or elusive performance of the state, which usually results in the exclusion of groups seeking to exercise their right to citizen participation. An example is the performance of the state in the management of the “Tia Maria” conflict, evidencing disarticulation and centralism, lack of impartiality, lack of legal and administrative capacities, loss of capacity to channel the demands of the population, loss of technical capacity, forced imposition of authority using police forces, and criminalization of protest (Ugarte, 2020).

Environmental conflicts in Peru and Latin America constitute a questioning of the role of public and private agents, which demand legal, planning and land use planning instruments, social licensing processes and institutional articulation (Fernández-Labbé, 2020).

Confirming what was reviewed in the preceding paragraphs, the departments of Loreto, Ancash, Cusco, Apurímac and Puno are located in the first quintile of social conflict, precisely those in which the extraction of natural resources is relevant. In the last quintile are Callao, Tumbes and others (see Figure 1), those departments with almost no extractive activity.

Broadening the spectrum, social conflicts have their origin mainly in inequality and social exclusion, distributive inequity, social exclusion, corruption, centralism, among others; they are structural problems, complex and with many edges and interconnections, which cannot be solved in Peru (Paredes and Encinas, 2020); therefore, they are often considered sources of instability for democracy in Peru. It is enough to review the violent changes of government in the country over the last 200 years: numerous coups d’état, numerous uprisings and social conflicts, numerous cases of corruption, etc. (Castillo et al., 2023).

### 3.2 Income inequality

Inequality is considered a consequence of the concentration of economic resources by small privileged groups, who use political strategies that allow them to curtail the rights of others so that the dispossessed do not have the resources and skills to mobilize politically. Certainly, this has a negative impact on institutional stability and democratic consolidation (Stezano, 2021). The unequal distribution of the national treasury, bordering on inequality, is probably one of the seeds of social conflict and democratic fragility. The vast majority of Peruvians consider that the government benefits large companies but evades or plunders wage earners in small and micro-enterprises, whether with direct or indirect taxes or administrative obstacles; therefore, “it should not be surprising that there is resistance and angry protests at certain times, as well as informality and illegality in the economy” (Francke, 2023). This unequal treatment, apparently a state policy, also manifests itself in the management of public finances.

Inequality, in terms of income distribution, is greater in the departments of Loreto, Callao, Ica, Cajamarca and San Martin; it is lower in Madre de Dios, Ucayali, Metropolitan Lima, Cusco, Tumbes, and Junín. The GINI coefficient of the population of the department of Loreto, the most unequal, is 0.48, and that of Junín, the lowest, is 0.28. Between the two, there is a distance of 71.4%, or almost three quarters.

Inequality is not necessarily characteristic of populations with particular development characteristics. The HDI of the departments in the first quintile, for example, in 2019 were very different: Loreto 0.483, Callao 0.640, Ica 0.600, Cajamarca 0.416 and San Martin 0.483; the same for those in the last quintile: Madre de Dios 0.614, Ucayali 0.484, Metropolitan Lima 0.707, Cusco 0.512, Tumbes 0.555 and Junín 0.511.

“Despite the progress in development achieved in recent decades, inequality continues to be one of the most present characteristics in the territory. Even Lima and the coastal departments, with higher HDI, have very marked interior heterogeneities” (PNUD, 2019). In this context, it is reasonable to attribute conflict to the strength of inequality, in its multiple forms (Ilizarbe, 2023), even beyond the strictly economic; indeed, social conflict between 2016 and 2023 was more relevant in two departments of the first quintile: Loreto and Cajamarca and two of the last quintile: Cusco and Junín (Figure 1).

### 3.3 Social exclusion

Another germ of social conflict is social exclusion, which manifests itself mainly in discrimination based on physical, linguistic, residence or other features of the indigenous population, which is relevant, if not the majority, in these Andean and Amazonian regions (Espinosa et al., 2021; Lovón and Palomino, 2022). Human exploitation and similar evils are not new, nor are conflicts between the rulers and the ruled; neither is the pretension of homogenization by making excessive use of political power, whether in the Inca, Viceroyalty or Republican States. In view of this, the uprisings are classic; for example, of the Chancas, the Collas, etc., in the Incanate; Tupac Amaru II in Cusco, Crespo y Castillo in Huánuco, the Zela brothers in Tacna and several others, at the end of the colony; in the Republican era, those led by Juan Bustamante, Teodomiro Gutiérrez, among others, in Puno; Hugo Blanco, in Cusco, and many, many others, throughout the national territory (Ruelas, 2019).

The forced process of homogenization, in general, responds to practices and interests alien to the Peruvian reality, were a strategic recreation of the viceregal order and are currently a replica of contemporary cultural colonization. An example of this is the homogenization approach to the cultural and social management of the recent environmental crisis disseminated, if not imposed, by governmental and non-governmental organizations and academia. The rationality and ancestral practices of environmental management of the Andean and Amazonian inhabitants do not always coincide with the academic, scientistic and legalistic garb promoted by modern environmentalists; rather, the exclusion, discrimination or deformation of the environmental, productive and social knowledge and interests of the indigenous population is notorious. This imposed culture, in turn, often stands as the source of conflicts within community organizations, in the loss of identity, even in the deterioration of productive and social processes (Alanoca and Apaza, 2018).

Social exclusion, more properly discrimination between 2016 to 2019, most frequently occurred in the departments of Puno, Tacna, Callao, Arequipa and Lima Region; and less in Apurímac, San Martín, Cajamarca, Ica, Loreto and Junín. The population that has reported the greatest discrimination corresponds to Puno: 28%; and the least, Apurimac: 4.42%. The social characteristics of the populations of these two departments are similar: significant indigenous population, majority practice of native languages, community social organizations, ancestral values, etc., in the various aspects of coexistence, productive organization, care of the environment, etc., potential discriminators are also present; however, for every five Puno residents who reported having suffered discrimination, only one in Apurimac did so. In the framework of the complexity of the problem, it is possible to rehearse some assumptions such as the incidence of the role assumed by local and regional authorities to eradicate it (Gallo, 2021), the greater resilience of the Apurimac population (Serra, 2020), the horizontal inequality of ethnic character (Puyana, 2018). The notorious recognition of discrimination in the populations of Tacna and Arequipa can be attributed to inter-regional and local migration (Incacutipa et al., 2022); in the case of Callao, to psychosocial factors resulting from the violent and criminal action of urban gangs (Azabal and Arruabarena, 2023); in the case of Lima Region, to the unattainable modernity of Metropolitan Lima. Probably, the declared resilience to discrimination of the population of San Martin and other departments can also be attributed to the factors mentioned for the population of Apurimac.

### 3.4 Public finance centralism

Political instability and social unrest hinder the process of orderly decentralization (Caballero, 2023). In this confusing context, central government officials appeal to political and administrative controls to direct the governance of subnational governments (Quispe, 2021). In effect, the initiatives directed at these governments are focused on improving management, digitized by the governing bodies of the administrative systems. The latter have resources, which, at most, channel them to provide technical assistance to subnational governments (Oliva, 2023). “Thirteen years after the launch of the decentralization process, the legal framework governing decentralization remains fragmented, complex and difficult to implement” reported (OCDE, 2016).

In this work, the literature review in this regard was confirmed. Between 2015 and 2022, the national government has executed 63.2% of the country’s public budget, regional governments 20.5% and local governments 16.3% (Gonzáles, 2022), confirming the finding of (Jurado and Tasayco, 2021).

In order of gradualness, the execution of the national government’s public budget, an indicator of the centralism of public finances, was higher in Metropolitan Lima, Callao, Madre de Dios, Lambayeque and Tumbes; it was lower in the departments of Cajamarca, Huancavelica, Apurímac, Ayacucho, Moquegua and Lima Region. The difference between the highest: Callao and Metropolitan Lima, each: 63.2% and the lowest, Ayacucho: 32.6%, is a ratio of 2 to 1. Moquegua: 29.7%, is even lower; but it is usually justified because it receives more mining canon, channeled through its regional and local governments.

The central administration of public finances by the Ministry of Economy and Finance (MEF), through the one-box system, digitizes budget management, as well as tax Research Topic and public indebtedness. Strictly speaking, regional and local governments are sounding boards for the MEF and other central government ministries; without the competencies and resources to design and manage their public policies endogenously (Jurado and Tasayco, 2021).

### 3.5 Corruption in subnational governments

The perception of corruption is one of the most frequent causes that are usually argued to delegitimize a national or subnational authority. One of the motivations that prompted Castillo to delegitimize himself was the “structural problem of corruption in various instances of the State” (Ilizarbe, 2023). It was also the main argument used to vacate Martín Vizcarra (Lovón et al., 2020). Almost all the presidents of the Republic, several high-level public officials of the central government, dozens of regional governors and mayors are involved in judicial proceedings for corruption. It seems that in Peru and in other Latin American countries, candidates for public office do it to appropriate public money. They pretend to fight corruption, but they are not interested in making it effective; rather, they try to ensure that legal institutions generate impunity, that corruption becomes professionalized, that it becomes an acceptable culture (Chavez de Paz, 2020); all to the detriment of those excluded from political power.

The list of damages that corruption causes to the social, economic and institutional system of a nation or part of it is very long: it deteriorates governance, the resources and potentialities necessary for sustainable development, the credibility of politicians, even of ordinary citizens who make use of public services; it reduces the efficiency and effectiveness of the allocation of public and private resources, of course, of public investment, delegitimizes the State, degrades and discredits the public function, of public and private suppliers of goods and services, and many others (Zavaleta, 2023).

As of September 2023, 55.6% of those surveyed consider corruption to be one of the country’s main problems (INEI, 2023). Fifty-eight percent of corruption cases are attributable to “contracting by regional and local governments” (Zavaleta, 2023), as controls and counterweights are weaker (Olaguivel et al., 2023); that is, 48% occur in the spheres of the national government. This study established that the perception of corruption in the department of Madre de Dios, according to its inhabitants, is 6.9%, the highest; it is 7.7 times higher than in Amazonas (see Figure 1). Metropolitan Lima and Madre de Dios are also the two regions in which the national government executed the largest public budget; on the other hand, three of the departments in the last quintile in terms of corruption according to their inhabitants are also those in the last quintile in terms of the execution of public spending by the national government. In synthesis, it could be deduced that corruption, at least in the execution of public spending, crosses all governmental levels. “The political and economic situation in Peru favors the emergence and spread of corruption at all levels of government and the anti-corruption fight, which originates from the interest of citizens, will be able to curb or mitigate it (Palacios et al., 2022).

With respect to social conflict, Puno and Ancash are in the first quintile; and in the same range in terms of perception of corruption. The three departments in the last quintile also coincide in the same range of social conflict and corruption; that is, there is a relative correspondence between the two variables.

Focusing our analysis to the sub-national environment, we review some recommendations for anti-corruption practices. With evidence from the years 2011–2014, (Mujica et al., 2017) revealed that corruption in regional and local governments are of three types: tithes for public bids, surreptitious negotiation with national and international suppliers through intermediaries, and linkage with networks associated with illegal markets and economies. These are induced by potential corrupters, availability of public resources and weak control systems. OECD considers that, among others, the common weaknesses of sub-national governments to undertake anti-corruption actions are the insufficient autonomy of their control systems, social organizations with weak capacities and citizen participation, insufficient practice of the New Public Governance, lack or insufficient application of digital technology to process and make information transparent (Ortiz, 2022). In this regard, the autonomous and unrestricted exercise of citizen participation is added, but with the philosophy and practices of social and community organizations, the valuation of the professional quality of public officials of sub-national governments to make use of technologies and strategies of good governance. Let the citizens of the regions and localities co-govern, control, reward or punish their authorities with their vote.

### 3.6 Revocation of subnational authorities

The revocations of authorities between 2016 and 2023, have only occurred in 2 years: 2017 and 2021. All of them were to authorities of district municipalities; the frequency was higher in Ancash, in 10 departments there was none of this process. The departments where there was more conflict between 2016 and 2023 were Loreto, Ancash, Cusco, Puno and Apurimac; it did not occur in Callao, with minimal frequency in Tumbes, Tacna, Ica, Ucayali and Madre de Dios. In Loreto, the average frequency is 20.5 conflicts per year, followed closely by the other departments.

Research on the revocation of sub-national authorities shows that they are one of the most relevant factors of social conflict, generally focused on a municipality. Law 26,300, referring to the right of citizen participation and control, contemplates this possibility (Congress of the Republic, 1994). This law, since its enactment, motivated more than five thousand sub-national authorities to undergo a recall process. Faced with this maelstrom, considered “perverse” due to the provocation of instability in the sub national governments, the Congress approved Law 30,315 (Congress of the Republic, 2015), modifying some articles of the original law, particularly that referring to the advancement of elections. The application of the latter drastically reduced the number of recall elections (Mendoza, 2019).

The actors that usually lead the recall are local political elites competing for political and economic power. They usually argue high citizen dissatisfaction due to poor governance, corrupt performance, inefficient, undemocratic, lack of transparency, unethical, inability to manage, submissive to political power. At the top of the justifications, are the aspiration of influential citizens for the use of direct democracy mechanisms, greater visibility of inequalities, poverty, exclusion, among other social ills (Abente, 2012; Mendoza, 2020; Quispe, 2021). The frequency of revocations is concentrated in departments with less social, economic, urban or institutional development or with high environmental risk, as occurs in the Andes and the Amazon or in the macro regions: center, south and east with respect to the coastal departments and the provinces of Lima and Callao (Coronel, 2019; Hausmann et al., 2022; Coyla et al., 2023). In the study horizon, two recall processes were carried out in 2017 there were 27 of these processes and 13 in 2021; all of them in rural district municipalities of hundreds but slightly more than a thousand electors.

### 3.7 Association of subnational social conflict and its inducers and between them

A review of the literature on the association between social conflict and its inducers revealed that a reduction in inequality from 0.49 to 0.43, in terms of GINI coefficients, corresponds to a reduction of about 18% in conflicts at the national level in the Republic of Chile (Pérez and Sandoval, 2020); that there is a territorial relationship between areas of social injustice and environmental injustice, through the recognition of points of interconnection between poverty and socio-environmental conflicts (Luna and Blancas, 2022); and, that greater decentralization contributes to better control of corruption in Mexico (Alvarado and Rodríguez, 2010). With this methodological justification, a matrix with Spearman correlation coefficients between social conflict and its inducers and between them was developed (see Figure 1).

Peruvian sub-national social conflict, in the horizon from 2016 to 2023, was positively and moderately related (0.40 to 0.69) with the recall of authorities: 0.492; that relationship is also positive but low (0.20 to 0.39) with income inequality: 0.296; negative but low (− 0.20 to −0.39) with the centralism of public finances; there is a very low and negative relationship (0.00 to - 0.19) with social exclusion: −0.131 and with corruption: −0.183.

These results allow us to validate, moderately and with reservations, the arguments put forward in the previous sections. The revocation of subnational authorities was a moderate source of social conflict in subnational governments, particularly in local district governments; along the same lines, although still on a smaller scale, was income inequality. On the other hand, contrary to what was predicted in the literature review, the greater execution of public spending by the national government, that is, the persistence in centralizing public finances, contributed to reducing, albeit irrelevantly, social conflicts. Possibly, this can be explained by the sense of dependence rooted in the regions and localities with respect to the power held by the national government in terms of controlling the distribution and transfer of the public budget (Escobar et al., 2021; Quispe, 2021; Caballero, 2023; Mendoza, 2023). The other two potential inducers, social exclusion and corruption, did not contribute to the reduction or increase of subnational social conflict over the study horizon.

The digression of the correlation between the inducers is intended to establish whether there is collinearity between them; but this is not the case. There is only one moderate correlation between corruption and social exclusion, the others are low or non-existent. However, with the few low or moderate coefficients of determination (R^2^), four regression models have been constructed; two, to attempt a plausible explanation of the frequency of subnational social conflicts from their inducers; the third and the fourth to attempt an explanation of social exclusion and centralism of public finances from the related variables.

With the exception of social conflict, the only inductor related to income inequality is the centralization of public finances, albeit at a low level and in a negative way. It seems to be a reverse transfer from the regions to the metropolis. For this reason, it should attract the attention of public policy makers, even more so of those who manage the execution of the public budget. Effective decentralization requires a real transfer of the public budget (Gonzáles, 2022).

Corruption contributes moderately to social exclusion and to a lesser extent fiscal centralism, the relationship with the other inducers is irrelevant. Because of the criminal nature of corruption, those who practice it seek to reduce its scope. One of the disasters that corruption entails is the isolation of corrupt rulers and public officials from the citizens they should serve, imposing authoritarian attitudes, including social exclusion to citizen participation (Zavaleta, 2023). Likewise, one of the inducers of corruption is precisely the access to considerable budgetary resources from the mining canon, FONCOR, FONCOMUN, etc. (Mujica et al., 2017).

The centralism of public finances is negatively and less than moderately related to the revocation of sub-national authorities and inequality; positively, but equally less than moderately related to social exclusion and corruption. One of the arguments usually used to promote the revocation of sub-national authorities is their inability to manage the budget, particularly to generate employment in the least favored districts or to engage in acts of nepotism, which further increase inequalities (Quispe, 2021). The relationship between centralism and corruption or social exclusion, or between corruption and other inducers, has been discussed in the preceding paragraphs.

Regression model No. 1 shows that the revocation of sub-national authorities determines 42.5% of social conflict. This determination is slightly reduced to 41.4% in model No. 2, when the predictor variables income inequality, centralism of public finances, and the recall of authorities are included (see Table 2). The significance of the recall in this model remains; on the other hand, the contributions of the other variables are not significant. Models No. 1 and No. 2 moderately explain the occurrence of social conflict mainly from the revocation of the authorities. Income inequality and the centralism of public finances only contribute to consolidate it.

**Table 2:** Linear regression models related to social conflict and its inducers.

	Model summary		ANOVA		Coefficients			
Model	R	R^2^ adjusted	F	Sig.	V. Dependent	Predictors	B	Sig.
1	0.664	0.425	27.594	0.000	Social conflict	Constant	3.805	0.001
						Revocation	0.724	0.000
2	0.683	0.414	9.017	0.000	Social conflict	Constant	12.927	0.080
						Inequality	−22.079	0.236
						Centralism	−0.001	0.991
						Revocation	0.705	0.000
3	0.559	0.283	10.657	0.000	Social exclusion	Constant	−1.415	0.677
						Corruption	0.219	0.008
						Centralism	1.153	0.002
4	0.492	0.218	9.898	0.000	Fiscal centralism	Constant	44.506	0.000
						Exclusion Social	0.425	0.000
						Inequality	−19.698	0.193
						Conflict	−0.340	0.005

Own elaboration with data from National Jury of Elections (2021), INEI (2024), Ministry of Economy and Finance (2024), Ombudsman’s Office (2024), and World Bank (2024).

Regression model No. 3 reveals that corruption and centralized public finances only account for 28.5% of the population’s perception of having suffered discrimination, the main element of social exclusion. It is to be assumed that there are other inducers or factors that also determine it. It is almost the same with the fact that social exclusion, inequality and social conflict only determine 21.8% of the relevance of the execution of public spending by the national government in the regions and localities or fiscal centralism.

## 4 Conclusion

The regression models analyzed in this study offer important insights into the factors that influence social conflict, the perception of discrimination, and the relevance of public expenditure execution at the subnational level in Peru.

The findings of this research reveal that there is a significant probability of 42.5% that social conflicts will occur in the case of a recall initiative of subnational authorities. However, when including additional variables such as income inequality and centralism of public finances, this probability decreases slightly to 41.4%. This decrease suggests a correlation between the dependence of regions and localities on central power in the distribution of resources and the possibility of social conflict.

In conclusion, the results obtained show that the perception of the population of suffering social exclusion due to corruption and centralism in public finances is low, registering 28.5%. However, this finding should not be interpreted as an absence of problems related to social exclusion, but rather as a sign that the direct perception of these factors may vary significantly among different communities and regions.

It is crucial to recognize that social exclusion can manifest itself in various forms and that its approach must take into account the particularities of each regional context. This implies the need to adopt multidisciplinary and intercultural approaches that consider not only corruption and centralism in public finances, but also other relevant factors that may contribute to social discrimination.

Inequality and social conflict influence 21.8% of fiscal centralism in the execution of public spending by the national government in the regions and localities.

A comprehensive strategy is needed to promote citizen participation, strengthen democratic institutions and foster inclusive economic and social development in all regions. Only through a holistic approach adapted to local realities will we be able to move towards building fairer and more equitable societies, where all citizens have equal opportunities and access to the resources necessary for their full development.

These results determine the need to review decentralization and resource distribution policies in order to more effectively address social conflict in the country. It is clear that the current dynamic of dependence on central power generates tensions that could be mitigated through a more equitable redistribution of resources and greater financial autonomy for subnational authorities. Ultimately, this research underscores the importance of designing policies that promote effective decentralization and citizen participation to prevent and manage social conflicts more efficiently.

## Data availability statement

The raw data supporting the conclusions of this article will be made available by the authors, without undue reservation.

## Author contributions

TL: Conceptualization, Formal analysis, Funding acquisition, Investigation, Methodology, Project administration, Writing – original draft, Writing – review & editing. MC: Conceptualization, Formal analysis, Investigation, Methodology, Supervision, Validation, Writing – original draft, Writing – review & editing. JR: Investigation, Methodology, Supervision, Validation, Writing – original draft, Writing – review & editing. JM: Data curation, Formal analysis, Investigation, Methodology, Software, Visualization, Writing – original draft, Writing – review & editing. GS: Conceptualization, Formal analysis, Investigation, Writing – original draft, Writing – review & editing. NV: Formal analysis, Investigation, Methodology, Visualization, Writing – original draft, Writing – review & editing.
